# Progress, critical challenges, and translational prospects of intranasal administration of botanical essential oils for the treatment of depression

**DOI:** 10.3389/fphar.2025.1748824

**Published:** 2026-02-09

**Authors:** Kexin Cheng, Hongyi Zhao, Kui He, Xiuwen Xia, Yi Wang, Shuyu Wang, Weihong Li

**Affiliations:** 1 School of Basic Medical Sciences, Chengdu University of Traditional Chinese Medicine, Chengdu, China; 2 School of Health Preservation and Rehabilitation, Chengdu University of Traditional Chinese Medicine, Chengdu, China; 3 Sichuan College of Traditional Chinese Medicine, Mianyang, China

**Keywords:** essential oil, depression, intranasal administration, psychiatric disorders, quality control

## Abstract

Depression is a highly prevalent and disabling psychiatric disorder worldwide. However, currently available first-line antidepressant therapies are frequently limited by delayed onset of action, a high incidence of adverse effects, and inconsistent therapeutic efficacy, underscoring the urgent need for alternative treatment strategies. Botanical essential oils are rich in volatile, lipophilic small-molecule metabolites and are characterised by multi-metabolite, multi-target synergistic regulatory properties. When combined with intranasal administration, these metabolites can exploit the nose-to-brain pathway to bypass the blood–brain barrier and achieve rapid central nervous system delivery, thereby emerging as a promising research focus in antidepressant therapy. In this study, a systematic review was conducted in strict accordance with the PRISMA guidelines, encompassing 40 relevant studies published between 2015 and 2025. We comprehensively summarised intranasal administration paradigms of botanical essential oils, including dosing regimens and temporal patterns, elucidated their core antidepressant mechanisms, and placed particular emphasis on their rapid modulatory effects on olfactory neural circuits and emotion-related brain regions. In addition, recent advances in novel delivery technologies—such as nanoemulsions, lipid-based nanocarriers, and mucoadhesive delivery systems—were critically reviewed for their roles in enhancing brain-targeting efficiency and formulation stability. Meanwhile, this review objectively evaluates major limitations in the current body of research, including insufficient experimental rigour, inconsistent administration parameters and quality control, inadequate systematic toxicological assessment, and substantial challenges associated with the standardisation of essential oil compositions. By integrating available experimental evidence with network pharmacology analyses, this work aims to provide a theoretical foundation and strategic guidance for future mechanistic studies, formulation optimisation, and the clinical translation of intranasal botanical essential oil–based interventions for depression.

## Graphical Abstract

**Figure:**
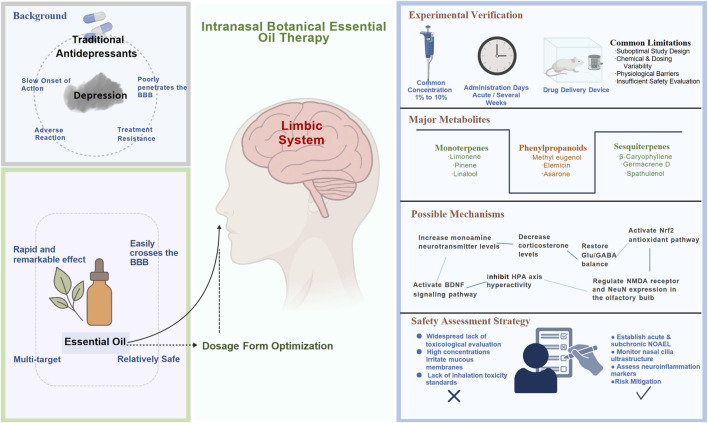

## 1 Introduction

Major Depressive Disorder (MDD), a common and highly disabling psychiatric condition, has shown a steadily rising global prevalence, placing a substantial burden on individuals, families, and society. Recent global crises—most notably the COVID-19 pandemic—have further elevated psychological stress across populations (Ma et al., 2020), intensifying the urgent need for effective, accessible, and patient-compliant antidepressant interventions. Although first-line pharmacotherapies currently used in clinical practice, particularly selective serotonin reuptake inhibitors (SSRIs), provide symptom relief for some patients, their limitations remain prominent: a delayed onset of action (typically requiring 2–4 weeks), frequent adverse effects (including weight gain, sexual dysfunction, and gastrointestinal discomfort), and treatment resistance in approximately 30% of patients (Saelens et al., 2025; Fiorillo, 2021; Zięba et al., 2021).These shortcomings collectively contribute to reduced adherence, prolonged disease courses, and an overall increase in disease burden. Accordingly, the search for alternative or adjunctive therapeutic strategies characterised by faster onset, fewer side effects, and novel mechanisms of action has become a major focus in psychopharmacology.

Against this backdrop, essential oils (EOs) derived from medicinal plants within various traditional medical systems—including Traditional Chinese Medicine, Ayurveda, and Mongolian medicine—have attracted growing scientific interest (Chen et al., 2023; Zhang et al., 2021). Contemporary pharmacological studies have increasingly demonstrated that the small-molecule lipid metabolites of essential oils—such as monoterpenes and sesquiterpenes—not only possess favourable volatility and membrane permeability but also exert synergistic, multi-metabolite, multi-target actions. These include modulation of central monoaminergic neurotransmission, enhancement of neurotrophic factor expression, suppression of neuroinflammation, and mitigation of oxidative stress (Spisni et al., 2023; Zhang et al., 2019; Zhong et al., 2024), collectively contributing to robust antidepressant-like effects in preclinical models. The standardised lavender-derived formulation “Silexan,” approved in Germany in 2009 for alleviating anxiety and depression-related symptoms, further supports the clinical translation potential of essential-oil-based therapeutics (Kasper et al., 2018). Moreover, combining botanical essential oils with intranasal administration represents a promising strategy to amplify their therapeutic potential. The nasal cavity serves not only as the organ for odour perception but also as a potential “nose-to-brain” drug delivery route (Du et al., 2017). This pathway capitalises on the close anatomical and functional connectivity between the upper olfactory epithelium and the brain: olfactory receptor neurons are directly exposed to the nasal mucosal surface, their axons traverse the cribriform plate to reach the olfactory bulb, and subsequently project to key emotion- and memory-related brain regions—including the amygdala, hippocampus, and prefrontal cortex. Through this route, therapeutics can bypass the blood–brain barrier, enabling rapid delivery from the peripheral environment to the central nervous system. Intranasal administration of essential oils thus offers potential advantages over traditional oral medications, mitigating slow onset and first-pass metabolism while providing a novel avenue for rapid-acting interventions in psychiatric disorders. However, the effective application of essential oils via this route is often hindered by their high volatility, poor water solubility, and instability. To overcome these physicochemical barriers, advanced delivery systems have been developed. Nanoemulsions are thermodynamically unstable but kinetically stable colloidal systems composed of oil, water, and surfactants, with droplet sizes typically ranging from 20 to 200 nm (Om et al., 2025). Their ultrafine droplet size increases surface area and enhances the solubility, stability, and bioavailability of lipophilic metabolites, making them advantageous for pharmaceutical and cosmetic delivery. Due to their small size, nanoemulsions exhibit improved penetration across biological barriers and provide rapid delivery of encapsulated actives (Akbari et al., 2024). Additionally, they offer optical transparency, high physical stability against creaming or sedimentation, and flexible formulation options for both hydrophilic and hydrophobic agents (Rahimnia et al., 2024). Therefore, summarizing these emerging technologies is critical for advancing the field.

Although several reviews have addressed the antidepressant potential of essential oils or discussed their delivery routes, an in-depth examination of methodological standardisation, advances in delivery technologies, and toxicological challenges over the past decade remains lacking. In contrast to previous reviews, the present work focuses specifically on the “nose-to-brain” pathway. It provides a detailed exposition of its anatomical and physiological underpinnings and adheres strictly to PRISMA guidelines in conducting a systematic review. We performed comprehensive literature retrieval, quality assessment, and methodological evaluation of 40 studies published between 2015 and 2025. We critically analyse essential-oil administration paradigms and core antidepressant mechanisms, and summarise emerging nanocarrier-based delivery technologies designed to enhance brain-targeting efficiency. At the same time, this review does not shy away from addressing the limitations and challenges in current research, including the frequent absence of positive controls, incomplete or imprecise reporting of administration parameters, insufficiently systematic toxicological assessments, and the inherent difficulties of standardising essential-oil composition. By objectively evaluating these issues—supplemented by preliminary network pharmacology-based predictions of shared essential-oil metabolites and their putative interaction networks—this review aims to provide informed guidance for the continued development and translational advancement of this field.

## 2 Anatomical and physiological foundations of the nasal-brain axis

The Nasal–Brain Axis refers to the direct anatomical and functional pathways connecting the nasal cavity to the central nervous system (CNS), a concept first proposed by Doty and colleagues (Consonni et al., 2022). This framework highlights that the nasal cavity is not merely a sensory gateway but an interface capable of modulating brain function at molecular, cellular, and dynamic levels. It mediates neuroimmune and pathological signal transmission and provides important insights into the pathogenesis, progression, and intranasal therapeutic modulation of neurodegenerative and psychiatric disorders.

### 2.1 Anatomical structure and function

**FIGURE 1:**
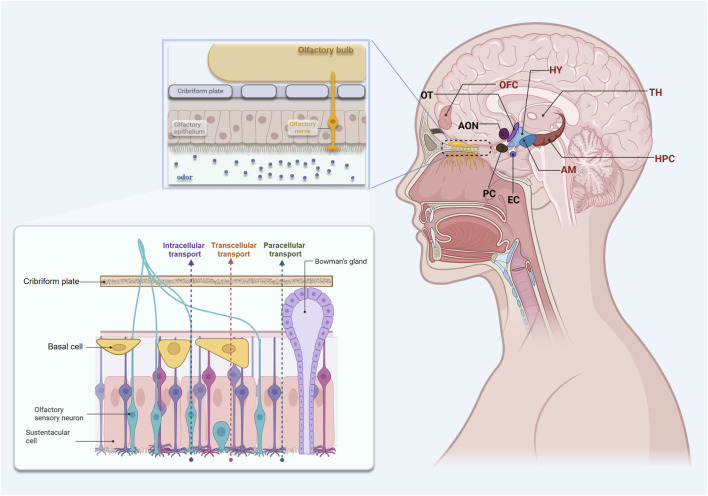
Schematic representation of the anatomical structures and drug delivery mechanisms of the nasal–brain pathway. (This figure depicts the major neural and transport pathways connecting the olfactory region of the upper nasal cavity to the CNS. The olfactory epithelium (OE) comprises bipolar olfactory sensory neurons (OSNs), sustentacular cells, basal cells, and Bowman’s glands. OSN dendrites extend into the mucosal surface to detect odorants, and their axons converge to form the olfactory fila, which traverse the cribriform plate to enter the olfactory bulb (OB). The OB relays olfactory signals to primary olfactory cortex regions—including the AON, PC, OT, and EC—and further to higher brain regions such as the OFC, HPC, AM, HY, and thalamus (TH). Drug molecules delivered intranasally cross the OE and its basement membrane to enter the lamina propria, from which they travel along olfactory nerve axons into the brain. The three principal drug transport mechanisms include itracellular transport, paracellular transport, and transcellular diffusion.).

Anatomically, the olfactory epithelium of the superior nasal meatus contains bipolar olfactory receptor neurons (ORNs), whose dendrites are directly exposed to the mucosal surface to detect inhaled odorants. Their axons bundle into the olfactory fila (fila olfactoria) and traverse the cribriform plate to reach the olfactory bulb (OB) (Nagayama et al., 2014; Rey et al., 2018). The OB serves as the first relay station, transmitting signals to the primary olfactory cortex (POC), which comprises the anterior olfactory nucleus (AON), piriform cortex (PC), olfactory tubercle (OT), and entorhinal cortex (EC). Neurons within the POC then project to higher-order olfactory and emotion-related brain regions, including the orbitofrontal cortex (OFC), hippocampus (HPC), hypothalamus (HY), and amygdala (AM) (Huart et al., 2013; Marin et al., 2023; Chen et al., 2026). This hierarchical organisation enables the integration of odor information with emotional and contextual cues (Figure 1). In contrast to other sensory modalities, olfactory information bypasses the thalamus and directly accesses emotion- and memory-related centres, providing the neural substrate for the rapid induction of emotional responses and the evocation of episodic memories (Wu et al., 2020; Miró et al., 2001).

### 2.2 Mechanisms of drug delivery via the nasal–brain axis

The nasal mucosa, particularly the olfactory epithelium—is highly amenable to drug delivery, and neuronally mediated transport constitutes an important route by which metabolites can reach the central nervous system (CNS). Intranasal drug transport relies primarily on the olfactory region in the upper nasal cavity, where the olfactory epithelium contains sustentacular cells, basal cells, and Bowman’s glands. After traversing the epithelial layer, drug molecules must cross the basement membrane to enter the lamina propria, from which they access the brain via olfactory nerve pathways that penetrate the cribriform plate. The axons involved in this process are ensheathed by olfactory ensheathing glia. Drug delivery through the nasal–brain axis operates via three principal mechanisms: an intracellular pathway, in which drugs are endocytosed by olfactory sensory neurons and transported slowly along axons; and two highly efficient extracellular pathways, including paracellular transport through tight junctions between epithelial cells and transcellular diffusion of lipophilic metabolites across cellular membranes. The latter two extracellular routes enable drugs to reach brain tissue within minutes and represent the primary mechanisms responsible for the rapid nasal–brain delivery observed in preclinical models (Qiu et al., 2025) (Figure 1).

The concurrent operation of these mechanisms helps explain the rapid CNS distribution frequently observed in animal and preclinical intranasal delivery studies. Because olfactory projections bypass the thalamus and directly engage key emotion-regulating structures—such as the amygdala, hippocampus, and prefrontal cortex—intranasal stimulation can swiftly modulate the excitation–inhibition balance and oscillatory coupling within neural circuits associated with depression. In the short term, such stimulation may shift emotional response thresholds; over longer timescales, it may reshape circuit function via neuroplastic processes, thereby exerting sustained effects on mood regulation.

## 3 Design and research landscape of antidepressant delivery via essential oils

In recent years, intranasal administration of botanical essential oils has attracted increasing attention as a novel therapeutic approach for depression. The core pathophysiology of depression involves neurotransmitter imbalances, neuroinflammation, impaired neuroplasticity, and dysfunction of the hypothalamic–pituitary–adrenal (HPA) axis. The olfactory system’s direct connectivity with the limbic–prefrontal circuitry provides a unique route for volatile botanical metabolites to access the central nervous system.

In this review, we systematically retrieved and evaluated studies published between 2015 and 2025 following the PRISMA 2020 guidelines, ultimately including 40 clinical and preclinical investigations. These studies encompassed three major categories: botanical extracts, single natural metabolites, and multi-metabolite formulations. Overall study quality varied, with some works lacking rigor in administration parameters, control selection, or toxicological assessment. Nevertheless, the majority provided valuable exploratory evidence.

This section systematically summarizes the key findings regarding intranasal intervention of botanical essential oils for depression, examines the underlying mechanisms and limitations, and highlights directions for future research.

### 3.1 Research methods

#### 3.1.1 Study selection

This systematic review was conducted in accordance with the latest Preferred Reporting Items for Systematic Reviews and Meta-Analyses (PRISMA) 2020 guidelines.Literature published between 2015 and 2025 was retrieved from PubMed and Web of Science databases using the following search strategies:

—PubMed: (“Essential oil” OR “Volatile oil” OR “Aromatic oil”) AND (“Intranasal” OR “Nasal” OR “Inhalation” OR “Nose-to-brain”) AND (“Depression” OR “Depressive disorder” OR “Antidepressant”)
—Web of Science: (“Essential oil” OR “Volatile oil” OR “Aromatic oil”) AND (“Intranasal” OR “Nasal” OR “Inhalation” OR “Nose-to-brain”) AND (“Depression” OR “Depressive disorder” OR “Antidepressant”)

Three researchers independently screened the titles, abstracts, and full texts of identified publications, removing duplicates and studies that did not meet eligibility criteria. All included studies investigated the intranasal administration of botanical agents (botanical extracts, formulations, or isolated natural metabolites) for the treatment of depression. Exclusion criteria were as follows: (1) review articles; (2) conference abstracts; (3) studies unrelated to the topic; (4) publications for which the full text was unavailable. Discrepancies among the three researchers were resolved through consultation with a corresponding author to achieve consensus.

#### 3.1.2 Data extraction and quality assessment

Three independent researchers conducted detailed data extraction from eligible studies, including: the name of the botanical agent, formulation, or metabolite and its metabolites; study subjects; dosage and concentration; method and timing of intranasal essential oil administration; primary assessment methods; proposed mechanisms underlying the antidepressant effects; and toxicological evaluation.

The methodological quality of each study was assessed using a nine-item checklist adapted from previous studies (Berger and Alperson, 2009; Yin et al., 2024):

1. Is the study objective clearly described?
2. Are the characteristics of study subjects adequately reported?
3. Is the grouping strategy described, appropriate, and justified?
4. Is the intranasal administration method sufficiently detailed?
5. Was a positive control used?
6. Are the results and data reported in sufficient detail?
7. Are the underlying mechanisms addressed and explained?
8. Do the results support the conclusions?
9. Was the toxicology of the intervention evaluated?

Scoring criteria: “2” = Yes; “1” = Partially Yes; “0” = No. The maximum possible score was 18 points.

#### 3.1.3 Study findings

**FIGURE 2:**
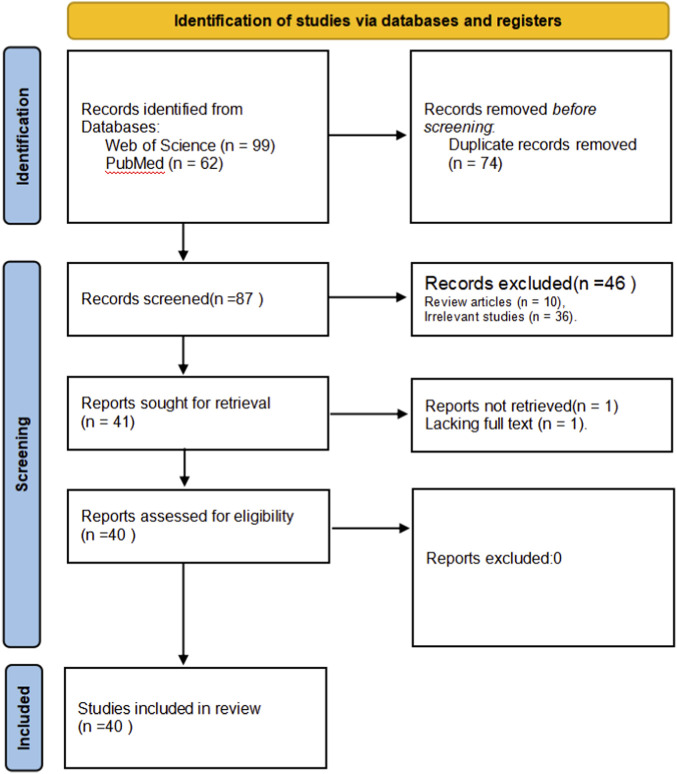
Systematic review flow diagram.

A total of 99 and 62 relevant studies were identified from Web of Science and PubMed, respectively, covering the period 2015–2025. Of these 161 studies, 74 duplicates, 10 review articles, 36 irrelevant studies, and 1 study lacking full text were excluded. Ultimately, 40 studies were included in this review (Figure 2). The quality scores of the included studies ranged from 12 to 18 points, with a mean score of 15.08 out of 18 and the most frequently observed score being 15. The corresponding journals’ impact factors (as of 2025) averaged 4.51, with a maximum of 9.5. Most studies clearly stated their objectives and provided sufficient information on study subjects, results, and appropriate conclusions (Table 1).

**TABLE 1:** Quality assessment scores for selected studies.

No.	Q1	Q2	Q3	Q4	Q5	Q6	Q7	Q8	Q9	Total score	References
1	2	1	1	2	2	2	2	2	0	14	Chen et al. (2023)
2	2	2	1	2	2	2	2	2	0	15	Ebrahimi et al. (2022)
3	2	2	1	2	2	2	2	2	0	15	Dos Reis et al. (2021)
4	2	2	1	1	2	2	2	2	2	16	Yin et al. (2024)
5	2	2	2	1	2	2	2	2	0	15	Khanh Nguyen et al. (2024)
6	2	2	2	2	2	2	2	2	0	16	Li et al. (2024)
7	2	2	2	1	2	2	1	2	0	14	Zhang et al. (2024)
8	2	1	2	1	2	1	1	2	0	12	Lai et al. (2024)
9	2	2	2	1	2	2	2	2	0	15	Wang et al. (2023)
10	2	2	2	1	0	2	2	2	0	13	Zhang S. et al. (2025)
11	2	2	2	2	0	2	2	2	0	14	Liang et al. (2018)
12	2	2	2	2	0	2	2	2	0	14	Isabel et al. (2019)
13	2	2	2	2	2	2	2	2	0	16	Kong et al. (2017)
14	2	2	2	1	2	2	1	2	0	14	Zhong et al. (2024)
15	2	2	2	2	2	2	1	2	0	15	Bagci et al. (2016)
16	2	2	2	2	2	2	1	2	0	15	Postu et al. (2022)
17	2	2	2	1	2	2	2	2	0	15	da Silva et al. (2025)
18	2	2	2	2	2	2	2	2	2	18	Chen et al. (2019)
19	2	2	2	2	2	2	2	2	0	16	Nguyen et al. (2024a)
20	2	2	2	2	0	2	2	2	0	14	Zhang et al. (2019)
21	2	2	2	2	2	2	2	2	2	18	Ueno et al. (2019)
22	2	2	2	2	2	2	1	2	0	15	Zhang Z-Y. et al. (2025)
23	2	2	2	2	0	1	1	2	0	12	Díaz-Cantón et al. (2024)
24	2	2	2	2	0	2	2	2	0	14	Kianpour et al. (2016)
25	2	2	2	2	2	2	2	2	0	16	Bahrie et al. (2022)
26	2	2	2	2	2	2	1	2	0	15	Nguyen et al. (2024b)
27	2	2	2	2	2	2	2	2	1	17	Aydin et al. (2016)
28	2	2	2	1	2	2	2	2	0	15	Chen et al. (2024)
29	2	2	2	1	2	2	2	2	2	17	Hashikawa-Hobara et al. (2019)
30	2	2	2	2	2	2	2	2	0	16	Tang et al. (2025)
31	2	2	2	2	2	2	2	2	0	16	Gong et al. (2023)
32	2	2	2	2	2	2	0	2	0	14	Tang et al. (2022)
33	2	2	2	2	2	2	1	1	0	14	Sur et al. (2020)
34	2	2	2	2	2	2	2	2	1	17	Saiyudthong and Mekseepralard (2015)
35	2	2	2	2	2	2	2	2	0	16	Ren et al. (2025)
36	2	2	2	2	2	2	2	2	0	16	Park et al. (2015)
37	2	2	2	1	2	2	2	2	0	15	Hritcu et al. (2016)
38	2	2	2	1	2	2	1	2	0	14	Ayuob et al. (2020)
39	2	2	2	1	2	2	2	2	0	15	Ayuob (2017)
40	2	2	2	1	2	2	2	2	0	15	Almohaimeed et al. (2021)

Q1:Is the study objective clearly described?

Q2:Are the characteristics of study subjects adequately reported?

Q3:is the grouping strategy described, appropriate, and justified?

Q4:Is the intranasal administration method sufficiently detailed?

Q5:Was a positive control used?

Q6:Are the results and data reported in sufficient detail?

Q7:Are the underlying mechanisms addressed and explained?

Q8:Do the results support the conclusions?

Q9:Was the toxicology of the intervention evaluated?

Among the 40 included studies, most employed essential oils extracted from natural plants, predominantly using distillation or supercritical CO_2_ extraction methods. Only a very small number involved metabolite formulations or isolated chemical monomers (fewer than three studies). This systematic review examines the concentrations, routes, and timing parameters used for intranasal essential oil administration, and synthesizes current evidence regarding their antidepressant mechanisms. On this basis, we summarize administration protocols that may serve as practical references and compile the included studies into a table to facilitate a comprehensive understanding of the research landscape, thereby providing guidance for the optimization of future experimental designs (Table 2).

**TABLE 2:** Overview of studies on intranasal administration of botanical essential oils for depression treatment.

No.	Botanical species (Family)	Sample description	Subjects	Dose range and MEC	Intranasal administration	Controls (Positive/Negative)	Model type	Primary assessment methods	Primary regulatory mechanism	Toxicity assessment	Reference	Impact factor (2025)
1	*Crocus sativus* L. (Iridaceae)	Commercial essential oil; GC-MS identified	*Mus musculus* (ICR)	2%; 4%; 6% (v/v,dissolved in fractionated coconut oil)	100 µL applied to filter paper for inhalation, twice daily, 4 h per session, for 20 consecutive days	Fluoxetine, administration/Fractionated coconut oil,inhalationadministratio	CUMS	Behavioural studies(OFT、SPT、TST、FST), haematoxylin and eosin staining, Nissl, WB; ELISA	MAPK-CREB1-BDNF signaling cascade: restores hippocampal neurons; elevates 5-HT/DA/GABA	No significant intergroup differences in organ coefficients	Chen et al. (2023)	5.4
2	*Lavandula stoechas* L. (Lamiaceae); *Matricaria recutita* L. (Asteraceae)	Commercial essential oil; no GC-MS report	human	1.50% (v/v)	3 drops nightly on cotton ball, 30 nights	No positive control/Distilled water inhalation	community-dwelling older adults	Questionnaire (DASS)	—	—	Ebrahimi et al. (2022)	2.2
3	*Lavandula angustifolia* Mill. (Lamiaceae)	Commercial essential oil; GC-MS identified	human	6% (v/v)	2 mL per bottle, one bottle nightly for 29 nights	Not applicable./Sunflower oil (placebo), same inhalation procedure	postmenopausal women with clinical insomnia	PSQI, ISI, HADS, MRS questionnaires; polysomnography (SOL, WASO, sleep efficiency, REM, etc.)	—	—	Dos Reis et al. (2021)	3.5
4	*Lavandula angustifolia* Mill. (Lamiaceae)	Commercial microencapsulated EO (β-cyclodextrin); GC-MS not performed	human	0.05 g/day	non-woven bag under pillow; Overnight bag-inhalation at bedtime, 4 weeks	No positive control/Empty bag placebo	Post-stroke depression patient model (clinical)	Questionnaire scales (PSQI, SDS, HAMD-17)	—	—	Yin et al. (2024)	3
5	*Citrus reticulata* Blanco. (Rutaceae)	Lab-made essential oil (steam-distilled); GC-MS identified	*Mus musculus* (ICR)	25 mg/kg; 12.5 mg/kg; 6.25 mg/kg (dissolved in 10 µL saline with 3% Tween-80)	Single intranasal instillation 10 μL, 30 min before behavioral tests	Memantine, intraperitoneal/Vehicle (3% Tween-80 saline) intranasal	Acute depression/anxiety model	Behavioural studies(TST、EPM); PC12/BV2 cells WST, LDH, ELISA, WB	Suppression of COX-2/PGE2 inflammatory pathway and protection against CORT neurotoxicity: increases cell viability, reduces LDH release	Lactate dehydrogenase (LDH) assay for olfactory bulb safety assessment	Khanh Nguyen et al. (2024)	4.8
6	*Lavandula angustifolia* Mill. (Lamiaceae)	Commercial essential oil; GC-MS identified	Sprague-Dawley rats	2.5% and 5% (v/v, diluted in distilled water, nebulization inhalation)	Twice daily, 30 min per session, for 7 consecutive days, nebulized inhalation	No positive drug/Model group inhaled distilled-water vapor	Alcohol-withdrawal-induced depression rat model	Behavioural studies(SPT、OFT、FST), 16S rDNA sequencing, untargeted metabolomics, hippocampal transcriptomics; ELISA	Downregulates TRPV4/Calml4 expression, suppresses hippocampal inflammatory cytokines, restores gut microbiota-hippocampus axis homeostasis	—	Li et al. (2024)	7.5
7	*Acorus tatarinowii* (Acoraceae); Panax ginseng (Araliaceae); Albizia julibrissin flower (Fabaceae)	Supercritical-extracted composite volatile oil; GC-MS identified	SD rats; PC12 cells	*In vivo* (rats): 0.05 mL/kg, 0.1 mL/kg; *In vitro* (PC12 cells): 20, 40, 80 μg/ml	perinasal inhalation, once daily, 28 days, Dropped on perinasal area, self-inhalation 30 min	Fluoxetine/Model group saline drops	*In vivo*:CUMS combined with orphan-rearing depression model; *In vitro*: Corticosterone	Behavioural studies, ELISA(SPT, FST, TST, OFT), HE, Nissl, IF; ELISA; WB; flow cytometry, cell counting kit-8 (CCK-8) assay, scratch wound healing assay	Activates cAMP-PKA-CREB pathway, elevates monoamine transmitters and BDNF, suppresses inflammatory cytokines, restores hippocampal neurons	—	Zhang et al. (2024)	8.3
8	*Curcumae Rhizoma*. (Zingiberaceae)	Steam-distilled (hydrodistillation); GC-MS identified	*C57BL/6* mice	1%; 2.5%; 5% (v/v, dissolved in normal saline)	Intransal administration	Fluoxetine/Normal saline	CUMS	Behavioural studies; *In vitro* antioxidant assays; *In vivo* antioxidant analysis; TEM; WB; IF; Nissl	Nrf2/HO-1/NQO1 pathway; Hippocampal oxidative stress levels; Mitochondrial ultrastructure of hippocampal neurons	—	Lai et al. (2024)	3.2
9	*Aquilaria sinensis* (Lour.) Spreng.(Thymelaeaceae)	Steam-distilled; GC-MS not performed	*Mus musculus*(KM)	2 μL; 4 μL; 8 µL (free vapor inhalation)	Filter paper placed in 50 × 50 × 40 cm incense box, once daily, for 7 consecutive days	Anxiety: Diazepam/mCPP model + air inhalation Depression: Paroxetine/CUMS model + air inhalation	Anxiety: mCPP; Depression: CUMS	Behavioural studies((LDET, OFT, TST, FST)), ELISA, WB	Bidirectional modulation of Glu/GABA system: anxiety↑GABAA, ↓Glu; depression↓GABAT/GRIN2B/GRM5, ↑GluR1/VGluT1, restoring E/I balance	—	Wang et al. (2023)	1.9
10	*Aquilaria sinensis* (Lour.) Spreng. (Thymelaeaceae)	Hydrodistilled agarwood essential oil (SAEO); Supercritical CO_2_ extracted agarwood essential oil (CAEO); GC-MS identified	*Balb/c* mice	SAEO inhalation: 4 μL, 8 μL; SAEO injection; CAEO inhalation: 4 μL, 8 µL	Heated aromatherapy at 80 °C, 1 h daily for 7 days	Paroxetine/Normal saline	LPS	Behavioural studies(OFT、TST、FST), ELISA, WB	Suppresses NF-κB/IκB-α inflammatory axis, upregulates BDNF/TrkB/CREB neurotrophic cascade: decreases pro-inflammatory cytokines, protects synaptic plasticity	—	Zhang S. et al. (2025)	4.8
11	*Liquidambar orientalis* Mill. (Altingiaceae)	Commercial essential oil; GC-MS not mentioned	*Mus musculus* (ICR)	10% (v/v, dissolved in 1% Tween-80 aqueous solution)	2 mL absorbed on cotton balls in 5 cm grid container, 10 min or 30 min once daily for 12 consecutive days	Negative: 1% Tween-80 inhalation	Acute restraint stress + Chronic Mild Stress (CMS)	Behavioural studies(OFT, SPT, TST, FST), ELISA	Reduction in ANG, TPO, IL-6, TNF-α; anti-inflammatory effects, regulation of vascular and platelet function	—	Liang et al. (2018)	1.7
12	*Lavandula angustifolia* Mill. (Lamiaceae)	Commercial essential oil; GC-MS identified	SD rats	2.5% (v/v, dissolved in 1% Tween-20 aqueous solution)	1 mL absorbed on cotton, 1 h inhalation daily for 14 consecutive days	Negative/1% Tween-20 inhalation	Chronic high-dose corticosterone	Behavioural studies (FST, SIT), BrdU/DCX immunohistochemistry, Sholl dendritic analysis, ELISA	↑BDNF and oxytocin serum levels; restores hippocampal/SVZ neurogenesis and dendritic complexity	—	Isabel et al. (2019)	2
13	*Chamaemelum nobile* (L.) All. (Asteraceae)	Commercial essential oil; GC-MS not mentioned	Wistar-Kyoto rats	Pure oil, 400 µL per session	400 µL continuously volatilized via electric aroma stone, 7 h daily for 14 consecutive days	Negative/no-oil exposure under identical housing	Genetic depression model	Behavioural studies(OFT, FST, SPT), iTRAQ proteomics, qRT-PCR (parvalbumin)	↑Oxidative phosphorylation and ↑hippocampal parvalbumin in PV-interneurons	—	Kong et al. (2017)	9.5
14	*Perilla frutescens* (L.) Britton. (Lamiaceae)	steam-distilled; GC-MS identified	SD rats	2 × 10^−3^; 4 × 10^−3^; 8 × 10^−3^ (v/v, dissolved in 1% Tween-80 aqueous solution)	Atomized inhalation, 60 min daily for 21 consecutive days	Fluoxetine/equal volume distilled water, intragastric administration	CUMS	Behavioural studies(SPT、FST), ELISA, IHC, WB, RT-PCR	Upregulation of monoamine neurotransmitter levels; Activation of the BDNF/TrkB signalling pathway	—	Zhong et al. (2024)	5.4
15	*Anthriscus nemorosa* (Bieb.) Spreng. (Apiaceae)	Commercial essential oil; GC-MS identified	Wistar-Kyoto rats	1%; 3% (v/v, vol/vol, direct inhalation)	Inhalation exposure to essential oil applied to filter paper, for 21 consecutive days	Diazepam,/Saline-Tween 80, inhalation	Scopolamine-induced amnesia	Behavioural studies(Y-maze, radial arm-maze, EPM, FST)	—	—	Bagci et al. (2016)	7.5
16	*Pinus halepensis* Mill. (Pinaceae)	Commercial essential oil; GC-MS identified	Wistar-Kyoto rats	1%; 3% (v/v, diluted in 1% Tween 80 solution for inhalation)	200 µL applied to vaporizer for inhalation, once daily, 15 min per session, for 21 consecutive days	Diazepam, Imipramine/1% Tween 80, inhalation	Aβ1-42-induced AD model	Behavioural studies (EPM, FST), oxidative stress assays, DNA fragmentation, ELISA, qRT-PCR	Nrf2/Keap1/ARE-CB2R axis: suppresses IL-1β, restores antioxidant enzymes, reduces DNA fragmentation	—	Postu et al. (2022)	3.9
17	*Myrcia sylvatica* (G.Mey.) DC (Myrtaceae)	Laboratory essential oil (hydrodistilled); GC-MS and GC-FID identified	*Mus musculus*(Swiss)	0.1%; 1%; 2% (v/v, 2 mL per cotton swab)	2 mL loaded on cotton swabs inside inhalation box, 5 min per session	Diazepam, Imipramine/Mineral water (p.o.) + 20% hydroalcoholic solution inhalation	Acute behavioral test	Behavioural studies, TLC, Ellman	Inhibition of acetylcholinesterase (AChE) activity	—	da Silva et al. (2025)	4.8
18	*Atractylodes lancea* (Thunb.) DC (Asteraceae), *Ambrosia artemisiifolia* Linn (Asteraceae), *Agastache rugosa* (Fisch. and C.A. Mey.) Kuntze (Lamiaceae), *Eupatorium fortunei Turcz* (Asteraceae), *Agastache rugosa* (Fisch. and C.A. Mey.) Kuntze (Lamiaceae), *Zanthoxylum bungeanum* Maxim. (Rutaceae), Amomum kravanh Pierre ex Gagnep. (Zingiberaceae), *Isholtzia ciliata* (Thunb.) Hyl. (Lamiaceae), *Acorus tatarinowii* Schott (Acoraceae), Kaempferia galanga L. (Zingiberaceae)	Volatile oil (hydrodistilled); GC-MS identified	SD rats	20.8 μL/kg; 5.2 μL/kg (intranasal)	Self-made atomizing box, daily inhalation for 4 weeks	Lavender volatile oi/saline, oral	CUMS + OBI	Behavioural studies (SPT, FST, OFT), ELISA	Restores DA/5-HT metabolism: elevates DA, 5-HT, HVA, 5-HIAA levels in mPFC and hippocampus	—	Chen et al. (2019)	5.4
19	*Perilla frutescens* (L.) Britton (Lamiaceae)	Laboratory essential oil (simultaneous distillation-extraction, SDE); GC-MS identified	*C57BL/6* mice	12.5 mg/kg; 25 mg/kg (dissolved in 3% Tween 80, intranasal)	10 µL/mouse, intranasal, 30 min before SDS induction or behavioral test, daily for 21 consecutive days	Memantine/3% Tween 80 in saline, intranasal	SDS	Behavioural studies (SIT, OFT, EPM, TST, FST), ELISA, WB; *In vitro* studies: Cell viability (WST), LDH assay, cytokine ELISA (TNF-α, IL-6), PAMPA-BBB	Modulation of corticosterone levels, hippocampal neurotransmitters (5-HT, GABA, NE) and ERK signalling pathways	Body weight changes, organ indices, serum biochemical parameters, and histopathological examination	Nguyen et al. (2024a)	5.4
20	*Citrus sinensis* (L.) Osbeck (Rutaceae)	Commercial essential oil (cold-pressed); GC-MS identified	*Mus musculus*(KM)	Filter paper loaded with 1 mL OEO/limonene for inhalation. Short-term: 1.5 h/day; Long-term: 24 h/day, for 5 days	Filter paper loaded with 1 mL OEO or 50 µL limonene placed inside cage, single daily exposure for 5 consecutive days	Fluoxetine/0.9% saline	CUMS	Behavioural studies(SPT、FST、OFT); serum lipid profile; ELISA; Western Blot; IF	Inhibits excessive HPA axis activation; elevates 5-HT, DA, and NE levels in prefrontal cortex and hippocampus; upregulates hippocampal BDNF and its receptor TrkB expression; improves dyslipidaemia	—	Zhang et al. (2019)	6.2
21	Rosa spp. (Rosaceae)	Synthetic 2-phenylethanol; no GC-MS of oil reported	*C57BL/6* mice	5% (v/v, dissolved in 1% Tween 80)	2 mL applied to cotton in stainless-steel container, 15 min inhalation before each test, single exposure per test day	Negative/1% Tween 80, inhalation	Normal mice	Behavioural studies (OFT, EPM, Y-maze, TST, PFST, SPT, grip strength test, cotton bud biting test)	Not specified	—	Ueno et al. (2019)	7.5
22	*Citrus aurantium* L. cv. Daidai (Rutaceae)	Steam-distilled essential oil; GC-MS identified	*C57BL/6* mice	100 μL; 200 μL; 400 μL(per 2 h inhalation session, undiluted)	CEO volatilized in 20 × 20 × 20 cm cage, 2 h once-daily inhalation for 28 consecutive days	Fluoxetine/CUMS group (no treatment inhalation)	CUMS	Behavioural studies (OFT, SPT, TST, FST, EPM), HE, Golgi-Cox, TEM, WB, ELISA; *In vitro* PC12 cell assays (cell viability, apoptosis, LDH, Ca^2+^, MDA)	Inhibition of cAMP/PKA/Grin2b signalling pathway; mitigation of excitotoxicity induced by glutamate synaptic hyperactivation; protection against hypothalamic synaptic deficits; restoration of HPA axis function	—	Zhang Z.-Y. et al. (2025)	5.4
23	*Litsea glaucescens* Kunth (Lauraceae)	Steam-distilled essential oil; GC-MS identified	*Mus musculus*(CD1)	20 μL(per 30 min inhalation session, undiluted)	20 μL volatilized in Petri dish inside 26 × 22 × 20 cm chamber, 30 min inhalation	Imipramine/Corn oil inhalation	Acute behavioral models	Behavioural studies (EPM, HBT, EC, OFT, FST), WB, ELISA	BDNF pathway activation in prefrontal cortex and hippocampus	—	Díaz-Cantón et al. (2024)	5.4
24	*Lavandula angustifolia* Mill. (Lamiaceae)	Commercial essential oil; no GC-MS data provided	human	3 drops, every 8 h, for 4 weeks postpartum	3 drops rubbed on palms then inhaled, every 8 h, for 4 weeks postpartum	Not applicable/Routine postpartum care only (no aromatherapy)	postpartum women	Questionnaire scales (EPDS; DASS-21)	—	—	Kianpour et al. (2016)	1.2
25	Rosa spp. (Rosaceae)	Synthetic 2-phenylethanol; no GC-MS of oil reported	*C57BL/6JR* mice	239 μL per session	239 µL impregnated on cotton gauze in closed box, 30 min/day, 15 consecutive days	Not applicable./Distilled water inhalation	Chronic corticosterone (CORT) oral administration	Behavioural studies; cFos immunohistochemistry (OB, amygdala, hippocampus, etc.)	Activation of neural circuits in the olfactory system	—	Bahrie et al. (2022)	7.5
26	*Pterocarpus santalinus* L.f. (Fabaceae)	Steam-distilled essential oil; GC-MS identified	*C57BL/6* mice	10 mg/kg; 20 mg/kg (dissolved in saline containing 3% Tween 80)	10 μL/mouse, once daily, 30 min before each SDS session, for 21 consecutive days	Memantine/3% Tween-80 saline	SDS	Behavioural studies (SIT, OFT, EPM, TST, FST); ELISA; Cellular assays	Modulation of the hypothalamic-pituitary-adrenal (HPA) axis, neurotransmitte;r systems (e.g., GABA, NE, 5-HT) and neuroinflammation (e.g., IL-1β, IL-6, TNF-α)	—	Nguyen et al. (2024b)	7.5
27	*Pimpinella peregrina* L. (Apiaceae)	Laboratory-prepared essential oil (hydro-distilled); GC-MS/GC-FID identified	Wistar rats	1%; 3% (v/v,dissolved in 1% Tween-80 saline)	200 µL placed in electronic vaporizer for inhalation, 15 min daily, for 21 consecutive days	Diazepam, Tramadol,Scopolamine/1% Tween-80 inhalation	Scopolamine-induced cholinergic amnesia	Behavioural studies (Y-maze, radial arm-maze, EPM, FST)	—	—	Aydin et al. (2016)	4.3
28	*Citri Reticulata* Pericarpium Viride. (Rutaceae)	Laboratory-prepared essential oil (steam-distilled); GC-MS identified	*C57BL/6N* mice	5%; 2.5% (v/v)	60 min continuous inhalation within 5-L sealed chamber, once daily for 10 consecutive days	Diazepam/oil-free chamber	CRS	Behavioural studies (OFT, EPM, FSPT), ELISA, HE, WB, IF, LFP, UPLC-MS,LFP in hippocampal DG area	Modulates Glu/NMDAR pathways in the olfactory bulb, inhibits glutamate toxicity, reduces NMDAR hyperactivation, promotes neurogenesis, improves cerebral blood flow, and regulates hippocampal theta oscillations	—	Chen et al. (2024)	5.4
29	*Chamaemelum nobile* (L.) All. (Asteraceae)	Commercial essential oil; GC-MS identified	*C57BL/6* mice	50 μL per cage (undiluted, neat oil)	50 μL applied to 1.5 mL tube with wire mesh, continuous exposure during 15-day stress period	Clomipramine/Saline	15-day restraint + water immersion stress	Behavioural studies, ELISA, IF, RT- PCR	Combined with Clomipramine, Promotes hippocampal neurogenesis, reduces serum corticosterone levels	—	Hashikawa-Hobara et al. (2019)	7.5
30	*Acorus calamus* L. (Acoraceae), *Santalum album* L. (Santalaceae), Citrus × limon (L.) Osbeck. (Rutaceae), Mentha piperita L. (Lamiaceae)	ultrasonic-assisted steam-distilled; GC-MS identified	SD rats	20; 100; 350 μL/mL (microemulsion stock)	30 min inhalation/session, once daily for 4 consecutive weeks	Diazepam/None	CUMS	Behavioural studies(SPT, OFT, EPM), Nissl, ELISA	Modulates monoamine and amino acid neurotransmitter systems, elevates hippocampal 5-HT and GABA levels, reduces glutamate content, whilst regulating key targets including ESR1, STAT3 and PPARG	Organ coefficient calculation and histopathological examination	Tang et al. (2025)	4.6
31	*Aquilaria sinensis* (Lour.) Spreng. (Thymelaeaceae)	*Mus musculus*(CD1)	*Mus musculus*(KM)	Essential oil (EO) 8 μL, agarwood powder (AFP) 1 g, agarwood incense stick (ALI) 1.25 g	Essential oil: 80 °C heating inhalation 1 h/session; Powder: 120 °C heating inhalation 5 h/session; Stick: combustion inhalation 1 h/session; once daily for 7 consecutive days	Diazepam, Paroxetine/no vehicle inhalation mentioned	Anxiety: m-CPP-induced; Depression: CUMS	Behavioural studies(OFT、LDE、TST、FST), ELISA, WB	Modulation of neurotransmitter secretion (5-HT, GABA, Glu) and expression of receptor and transporter proteins (GluR1, VGluT1)	—	Gong et al. (2023)	—
32	*Citrus reticulata* Blanco. (Rutaceae)	lab-scale steam-distilled; GC-MS identified	*Mus musculus*(KM)	0.625; 1.25; 2.5 mL/kg (neat oil for inhalation)	1 h inhalation/session, once daily for 7 consecutive days	Fluoxetine/Vehicle (1% DMSO, intraperitoneal injection	Reserpine-induced depression	Behavioural studies(FST, TST); Nissl; IHC; WB; RT-qPCR	Modulation of HPA axis function, upregulation of GR, BDNF and 5HT-1A receptor expression	—	Tang et al. (2022)	1.4
33	*Hypericum scabrum* L. (Hypericaceae)	​	Wistar rats	1%; 3% (v/v, dissolved in 1% Tween 20 aqueous solution)	200 μL placed in vaporizer, 15 min/session, once daily for 21 consecutive days	Diazepam, Tramadol/saline-Tween 20 inhalation	Scopolamine-induced dementia/anxiety-depression	Behavioural studies(EPM, FST)	—	—	Sur et al. (2020)	2.1
34	*Citrus bergamia* Risso. (Rutaceae)	Commercial essential oil; GC-MS identified	Wistar rats	2.5% (w/w, water/oil emulsion)	7 min daily inhalation in 36 × 30 × 29 cm chamber, 14 consecutive days	Fluoxetine/0.9% saline	CRS	Behavioural studies (FST), ELISA	None	—	Saiyudthong and Mekseepralard (2015)	—
35	Citrus × aurantium L. (Rutaceae)	Laboratory-extracted volatile oil; GC-MS identified	SD rats	1.335 μL/kg; 0.665 μL/kg; 0.335 μL/kg	0.335–1.335 μL/kg, tid, for 17 consecutive days	Fluoxetine/control group was drug-free	CUMS	Behavioural studies (FST, OFT), ELISA, IHC, WB, qPCR	upregulates GABA_A α1/γ2 receptors and mRNA; rebalances Glu/GABA ratio	Assessment of local irritation in nasal mucosa via H&E staining	Ren et al. (2025)	0.9
36	*Asarum heterotropoides* F. Schmidt. (Aristolochiaceae)	Laboratory essential oil (n-hexane extract, vacuum-concentrated); GC-MS identified	*Mus musculus* (ICR)	0.25 g; 0.5 g; 1.0 g; 2.0 g (neat, placed in uncapped tube)	Whole-body inhalation exposure	Fluoxetine/Saline inhalation	Acute forced swimming and tail suspension stress	Behavioural studies (FST, TST), IHC	Attenuation of stress-induced increases in CRF and TH, and decreases in 5-HT	—	Park et al. (2015)	3.4
37	*Juniperus communis* L. (Cupressaceae)	Laboratory volatile oil (hydro-distilled); GC-MS/FID identified	Wistar rats	1%; 3% (v/v, dissolved in 1% Tween 80)	200 µL placed on electronic vaporizer for whole-body inhalation, 60 min daily, 21 consecutive days	Diazepam, Tramadol/0.9% saline +1% Tween 80 inhalation	Aβ(1–42) i.c.v.-induced Alzheimer’s disease model	Behavioural studies (EPM, FST), enzyme activity assays	Antioxidant effects mitigate Aβ(1–42)-induced oxidative stress	—	Hritcu et al. (2016)	2
38	*Ocimum basilicum* L. (Lamiaceae)	Laboratory essential oil (hydro-distilled); GC-MS identified	Swiss albino mice	Not mentioned	Whole-body exposure in 32 × 24 × 32 cm odor-isolated acrylic box, 2 weeks, daily sessions	Fluoxetine intragastric/Amyl acetate 5% inhalation	CUMS	Behavioural studies (FST, EPM); ELISA, RT-PCR; HE; IHC	Upregulation of GFAP and Ki67 gene expression in the primary olfactory bulb enhances neurogenesis; downregulation of caspase-3 gene expression reduces apoptosis	—	Ayuob et al. (2020)	3.2
39	*Moschus moschiferus* (Animal-derived)	Natural musk secretion; GC-MS identified	Swiss albino mice	1.0% (v/v, dissolved in propylene glycol)	2.5 mL applied to cotton balls in acrylic box (32 × 24 × 32 cm), 15 min daily, 14 consecutive days	Fluoxetine intragastric/Amyl acetate 5% inhalation	CUMS	Behavioural studies (FST, EPM, OFT); ELISA, RT-PCR; HE staining; IHC	Upregulation of GR and BDNF expression in the hippocampus	—	Ayuob (2017)	1.7
40	*Moschus moschiferus* (Animal-derived)	Natural musk secretion; GC-MS identified	Swiss albino mice	1.0% (v/v, dissolved in propylene glycol)	2.5 mL applied to cotton balls in acrylic box (32 × 24 × 32 cm), 15 min daily, 14 consecutive days	Fluoxetine intragastric/Amyl acetate 5% inhalation	CUMS	Behavioural studies (FST, EPM); ELISA, RT-PCR; HE; IHC; oxidative stress markers	Anti-inflammatory and antioxidant mechanisms: reduces serum pro-inflammatory cytokines and MDA, enhances antioxidant enzymes activity; inhibits MOB apoptosis, promotes cell proliferation, improves neuronal structure in olfactory bulb	—	Almohaimeed et al. (2021)	2.9

### 3.2 Systematic analysis of administration regimens in essential oil–based antidepressant studies

#### 3.2.1 Summary of dosage-concentration patterns

A comprehensive review of 40 studies published between 2015 and 2025 indicates that essential oil dosage concentrations used for antidepressant purposes are highly variable but generally characterised by low exposure levels. Commonly reported units include percentages (%), as well as mass- or volume-normalised metrics (e.g., mg/kg, μL/kg). Several studies administered fixed daily volumes (e.g., 2 μL, 4 μL, 8 μL) or employed non-standardised metrics such as duration–drop count combinations. With respect to dosage design strategies, most studies adopted escalating concentration series (e.g., 2%, 4%, 6%) to enable more accurate evaluation of dose–response relationships. Overall, percentage concentrations typically ranged from 1% to 10%, whereas bodyweight-adjusted dosages largely fell within the range of several milligrams or microlitres per kilogram. These findings suggest that essential oils can exert their expected biological effects even at relatively low exposure levels.

#### 3.2.2 Summary of administration methodologies

To ensure stable respiratory exposure to volatile essential oil metabolites, enclosed chambers are widely employed as the core apparatus for administration. Three primary administration formats are commonly reported. The first, and most frequently used, is the enclosed chamber coupled with a nebuliser, which utilises specialised equipment to generate homogeneous aerosols and thereby achieve higher-concentration inhalation exposure. The second approach, enclosed chambers equipped with essential-oil–impregnated cotton balls, relies on natural volatilisation within a sealed environment and represents a simple form of passive inhalation. The third approach, intranasal instillation, involves delivering minute volumes of essential oil solutions directly into the nasal cavity, allowing for precise dose control. Additional auxiliary methods—including filter paper sniffing and heat-assisted volatilisation—are also reported. Collectively, these methodologies demonstrate a preference for enclosed systems to minimise environmental confounders and maintain a practical balance between dosing precision and operational feasibility.

#### 3.2.3 Summary of administration timing patterns

Analysis of administration timing reveals protocols spanning from a few minutes to several weeks, depending on experimental objectives and the physicochemical properties of the essential oils used. Broadly, these can be categorised into acute and chronic administration paradigms. Acute administration is typically designed to evaluate immediate effects, such as behavioural assessments conducted 30–60 min after dosing to detect rapid anxiolytic or sedative responses. In contrast, chronic administration is employed in long-term exposure studies or chronic disease models, often involving daily 30-min inhalation sessions over 12 or 28 consecutive days to evaluate antidepressant-like effects. Once-daily administration is the most common regimen, typically sustained for 7–28 days, facilitating the assessment of cumulative or long-lasting effects.

### 3.3 Mechanisms underlying the antidepressant effects of botanical essential oils

#### 3.3.1 Regulation of neurotransmitters and enhancement of neuroplasticity

A principal mechanism through which essential oils exert antidepressant effects involves the modulation of central neurotransmitter levels and the enhancement of neural plasticity. Evidence indicates that essential oils can elevate monoamine neurotransmitters—including serotonin (5-HT), dopamine (DA), and norepinephrine (NE)—and restore the balance between glutamate (Glu) and γ-aminobutyric acid (GABA), thereby improving synaptic transmission. For example, saffron essential oil significantly increases serum levels of 5-HT, DA, BDNF, and GABA, and ameliorates hippocampal neuronal injury and synaptic dysfunction by activating the MAPK–CREB1–BDNF signalling pathway (Chen et al., 2023). Active metabolites from Citrus spp. essential oils (CBEO) interact with neuroactive receptors and key signalling pathways, demonstrating strong binding affinity for depression-associated target proteins. Animal studies further confirm their rapid antidepressant and anxiolytic properties (Khanh Nguyen et al., 2024). In addition, Chang Shen Hua volatile oil (CSHVO) activates the cAMP–PKA–CREB pathway, enhances BDNF expression, and mitigates neuroinflammation, collectively promoting neuronal survival and synaptic plasticity (Zhang et al., 2024).

#### 3.3.2 Multisystem synergistic defence against stress, inflammation, and oxidative damage

Depression is tightly linked to dysregulation of the stress response system, heightened inflammatory signalling, and oxidative injury. Essential oils exhibit notable protective effects across these interrelated pathological domains. Multiple studies have shown that essential oils can suppress hyperactivation of the hypothalamic–pituitary–adrenal (HPA) axis, reduce corticosterone (CORT) levels, and facilitate the restoration of stress-response homeostasis. For instance, sweet orange essential oil (OEO) and its major metabolite limonene reverse behavioural deficits in CUMS-exposed mice, attenuate HPA axis overactivity, and upregulate DA, NE, and BDNF–TrkB expression (Zhang et al., 2019). Regarding anti-inflammatory actions, lavender essential oil (LEO) reduces hippocampal pro-inflammatory cytokines and modulates gut microbiota composition and metabolites, thereby improving gut–brain axis function and implicating both immune and metabolic regulation in its antidepressant mechanism (Li et al., 2024). In terms of oxidative stress defence, Curcuma rhizome volatile oil (CRVO) activates the Nrf2/HO-1/NQO1 antioxidant pathway, increases the activities of SOD, CAT, and GSH, and decreases ROS and MDA levels, ultimately alleviating CUMS-induced neuronal and mitochondrial damage (Lai et al., 2024).

#### 3.3.3 Rapid modulatory advantages of the olfactory neural circuit

Unlike orally administered pharmaceuticals, essential oils can rapidly access the central nervous system through the olfactory pathway, acting directly on olfactory bulb–limbic neural circuits to produce swift emotional regulation. This route exerts immediate influence on neurotransmitter release, synaptic dynamics, and neurogenesis within key emotion-related brain regions, including the hippocampus and amygdala. Consequently, the olfactory pathway is emerging as a promising therapeutic target for depression and may help catalyse a paradigm shift in the field. Studies demonstrate that green tangerine essential oil mitigates anxiety-like behaviours by modulating NMDAR and NeuN expression in the olfactory bulb, normalising glutamate and corticosterone levels, restoring 5-HT, and enhancing hippocampal neuronal firing activity. Basil essential oil promotes neurogenesis and neuronal resistance to apoptosis within the olfactory bulb by upregulating Ki67 and GFAP and downregulating Caspase-3, thereby improving depressive-like behaviours in CUMS mice (Zhang Z-Y. et al., 2025).These studies highlight the olfactory bulb not just as a sensory organ, but as a potential critical therapeutic target. However, current evidence relies heavily on histological markers,and direct electrophysiological evidence linking olfactory stimulation to rapid limbic circuit modulation remains sparse, representing a key gap for future mechanistic research (Li Q. et al., 2023; Rottstaedt et al., 2018).

### 3.4 Summary of chemical metabolites in antidepressant botanical essential oils and network pharmacology analysis

#### 3.4.1 Standardisation and statistical analysis of chemical metabolites in medicinal essential oils

This study systematically compiled the chemical composition of 42 medicinal essential oils through an extensive literature survey. For each oil, the primary metabolites reported in the literature (typically the top three; GC-MS data unavailable for a few oils) were selected as representative metabolites, yielding 118 chemical metabolites in total (Table 3). To ensure consistency in statistical analyses, metabolite names were standardised across sources. metabolites with differing nomenclature across studies or analytical systems were unified under a standard designation. For example, D-limonene, Limonene, and D-Limonene were standardised as Limonene; 1,8-Cineol and Eucalyptol were standardised as Eucalyptol; and Myrcene and β-Myrcene were standardised as Myrcene. Ultimately, 83 major metabolites were retained for subsequent analyses.Frequency statistics were then performed across all essential oil metabolites. The 16 most frequently occurring metabolites were: Limonene, Linalool, Linalyl acetate, Myrcene, Eucalyptol, α-Pinene, β-Pinene, β-Caryophyllene, Spathulenol, Methyl eugenol, Asarone, γ-Terpinene, Germacrene D, Elemicin, Menthone, and Perillaldehyde.

**TABLE 3:** Principal chemical metabolites of essential oils.

Essential oil name	Principal metabolites (by GC-MS)	References
Crocus sativus L.(Iridaceae)	Safranal, α-Isophorone, β-Isophorone	Eghbali et al. (2023)
Lavandula stoechas L.(Lamiaceae)	Linalyl acetate, Linalool, Lavandulyl acetate	Xu et al. (2023)
Matricaria recutita L.(Asteraceae)	(E)-β-farnesene, chamazulene, α-bisabolol oxide B	Lu et al. (2024)
Citrus reticulata Blanco. (Rutaceae)	D-limonene, c-terpinene, Methyl methanthranilate	Duan et al. (2016)
Panax ginseng (Araliaceae)	β-Panasinene, ginsinene, α-isocomene	Kim et al. (2022)
Acorus tatarinowii (Acoraceae)	β-Asarone, α-Asarone, α-Cadinol	Huang et al. (2024)
Curcumae Rhizoma.(Zingiberaceae)	Curzerene, Curdione, Germacrone	Lai et al. (2024)
Aquilaria sinensis (Lour.) Spreng.(Thymelaeaceae)	Isoaromadendrene epoxide, Agarospirol, β-Guaiene	Wang et al. (2018)
Liquidambar orientalis Mill.(Altingiaceae)	Styrene, Benzene propanol, Cinnamyl alcohol	Cengiz Baloglu et al. (2023)
Ambrosia artemisiifolia Linn(Asteraceae)	Germacrene D, β-Pinene, Limonene	Han et al. (2021)
Agastache rugosa (Fisch. and C.A.Mey.) Kuntze(Lamiaceae)	Pulegone, Estragole, Menthone	Hou et al. (2022)
Chamaemelum nobile (L.) All.(Asteraceae)	β-Oplopenone, Spathulenol, Himachalene	El-Assri et al. (2025)
Perilla frutescens (L.) Britton(Lamiaceae)	Perillaldehyde, 1,4-Cineole, Acetaldehyde diethyl acetal	Zhong et al. (2021)
Anthriscus nemorosa (Bieb.) Spreng.(Apiaceae)	Myrcene, γ-Terpinene, Germacrene D	Baldassarri et al. (2021)
Pinus halepensis Mill.(Pinaceae)	Myrcene, (Z)-β-Caryophyllene, α-Pinene	Djerrad et al. (2017)
Myrcia sylvatica (G.Mey.) DC(Myrtaceae)	β-Selinene, E-Calamenene, ar-Curcumene	da Silva et al. (2025)
Atractylodes lancea (Thunb.) DC(Asteraceae)	β-Eudesmol, hinesol, atractylone	Qiu et al. (2023)
Perilla frutescens (L.) Britton(Lamiaceae)	Elemicin, Apiol, Perillaldehyde	Nguyen et al. (2024a)
Citrus sinensis (L.) Osbeck(Rutaceae)	D-limonene, Thujene, Myrcene	El Hachlafi et al. (2024)
Rosa spp. (Rosaceae)	2-phenylethanol	Ueno et al. (2019)
Citrus aurantium L. cv. Daidai(Rutaceae)	Linalool, Linalyl acetate, D-limonene	Zhang Z-Y. et al. (2025)
Litsea glaucescens Kunth (Lauraceae)	1,8-Cineol (eucalyptol), α-Pinene, β-Pinene	Díaz-Cantón et al. (2024)
Eupatorium fortunei Turcz(Asteraceae)	Caryophyllene, β-Bisabolene, Spathulenol	Cui et al. (2002)
Santalum album L.(Santalaceae)	α-santalol, β-santalol, (Z)-α-trans-bergamotol	Mohankumar et al. (2019)
Pterocarpus santalinus L.(Fabaceae)	(+)-Ledene, Cedrol, Isoaromadendrene epoxide	Nguyen et al. (2024b)
Pimpinella peregrina L.(Apiaceae)	α-bergamotene, grandlure IV, methyl eugenol	Castagliuolo et al. (2025)
Citri Reticulata Pericarpium Viride.(Rutaceae)	4-Carene, p-Cymene, 3-Carene	Yi et al. (2015)
Citrus × limon (L.) Osbeck.(Rutaceae)	α-Pinene, β-Pinene, D-Limonene	Kaskoos (2019)
Acorus calamus L.(Acoraceae)	Asarone, Calarene, 2-Methoxy-3-allyl phenol	Kongkham et al. (2024)
Hypericum scabrum L.(Hypericaceae)	α-Pinene, α-Thujene, γ-Terpinene	Ergin et al. (2022)
Citrus bergamia Risso.(Rutaceae)	Limonene, Linalyl acetate, Linalool	Sawamura et al. (2006)
Citrus × aurantium L.(Rutaceae)	Linoleic acid, Stearic acid, Heptadecanoic acid	Li Y. et al. (2023)
Asarum heterotropoides F.Schmidt.(Aristolochiaceae)	Methyleugenol, Elemicin, 1,2,3-Trimethoxy-5-methylbenzene	Hu et al. (2025)
Juniperus communis L.(Cupressaceae)	α-Pinene, Germacrene D, β-Caryophyllene	Falasca et al. (2016)
Ocimum basilicum L.(Lamiaceae)	Methyl eugenol, α-Cubebene, Nerol	Özcan and Chalchat (2002)
Zanthoxylum bungeanum Maxim.(Rutaceae)	β-Myrcene, Linalool, D-Limonene	Feng et al. (2024)
Moschus moschiferus(Animal-derived)	Muscone, 2-Methylhexadecan-1-ol, Palmitic acid	Ding et al. (2022)
Amomum kravanh Pierre ex Gagnep.(Zingiberaceae)	Eucalyptol, β-Pinene, Limonene	Katerina et al. (2023)
Kaempferia galanga L.(Zingiberaceae)	Ethyl p-methoxycinnamate, Methyl cinnamate, Carvone	Tewtrakul et al. (2005)
Mentha piperita L.(Lamiaceae)	Menthol, Menthone, Eucalyptol	Abbas et al. (2024)

#### 3.4.2 Network pharmacology analysis of the potential mechanisms underlying the antidepressant effects of essential oils

Existing reviews on the antidepressant effects of essential oils largely adhere to traditional pharmacological frameworks, emphasising metabolite identification and effect summarisation. Due to limitations in experimental feasibility and cost, mechanistic studies remain preliminary and insufficient for elucidating synergistic interactions among multiple metabolites. While conventional studies can reveal the activity of individual metabolites, they fall short in characterising the multi-target, multi-pathway effects that typify essential oil pharmacology.

We therefore propose that integrating network pharmacology into essential-oil research enables a more comprehensive and systems-level evaluation of their antidepressant potential. This approach facilitates the construction of global interaction networks and helps compensate for the limitations of single-metabolite studies. It also possesses predictive utility: by integrating data from multiple databases, network analysis can identify key molecular targets and signalling pathways that recur across essential oils and are closely associated with the pathophysiology of depression. This strategy not only provides a theoretical basis for elucidating shared antidepressant mechanisms but also informs subsequent experimental validation and drug-development efforts.

**FIGURE 3:**
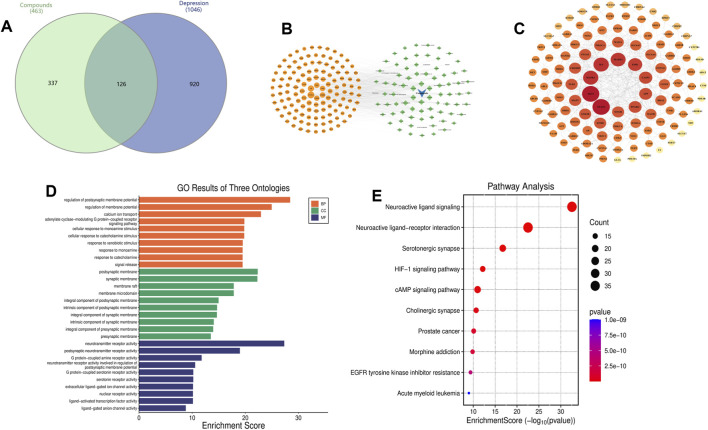
Network pharmacology analysis of essential oil metabolites against depression. **(A)** Venn diagram showing the overlap between metabolite-associated targets and depression-related targets. **(B)** Metabolite–disease–target network; triangles denote metabolites, circles represent core targets, and rhombuses indicate metabolites. **(C)** Protein–protein interaction (PPI) network of potential therapeutic targets (116 nodes, 380 edges); node color and size correlate with degree values. **(D)** GO functional enrichment analysis (biological process, cellular component, and molecular function). **(E**) KEGG pathway enrichment analysis.

SMILES identifiers for 83 major metabolites from 42 essential oils were retrieved from the PubChem database (https://pubchem.ncbi.nlm.nih.gov/). SwissTargetPrediction (http://www.swisstargetprediction.ch/) was used to predict molecular targets, and results were merged and deduplicated to generate a comprehensive target set. Depression-related targets were collected from the GeneCards and OMIM databases; after screening (excluding GeneCards targets with a relevance score <3.8), a total of 1,046 disease-associated targets were obtained. Metabolite-associated and disease-associated targets were compared using an online Venn diagram tool (https://www.interactivenn.net/index2.html) to determine overlapping targets, which were visualised in a Venn diagram (Figure 3A). A “metabolite–disease–target” network was subsequently constructed in Cytoscape. In the resulting visualisation, triangles denote metabolites, circles represent core targets, and rhombuses indicate metabolites. The top three core metabolites identified were Asarone, Linoleic acid, and Elemicin (Figure 3B). The 126 potential therapeutic targets were imported into the STRING database (https://www.stringdb.org/) for protein–protein interaction (PPI) network construction. Analyses were conducted using *Homo sapiens* as the organism, with a confidence score >0.7 and disconnected nodes removed. The PPI data were imported into Cytoscape to calculate topological parameters including degree, betweenness centrality, and closeness centrality. The top ten targets ranked by degree were designated as core nodes. The final network consisted of 116 nodes and 380 edges, with an average degree of 6.793. Node colour and size correlated with degree values, where darker and larger nodes indicated higher target connectivity (Figure 3C). GO functional annotation and KEGG pathway analysis were performed using the MicroBioinformatics platform (https://www.bioinformatics.com.cn/), and results were visualised accordingly.

GO enrichment analysis (Figure 3D) revealed that the identified genes were significantly enriched in biological processes (BP) associated with synaptic activity and neural signal regulation, such as postsynaptic membrane potential regulation, calcium ion transport, and responses to monoamines and catecholamines. These processes are intimately linked to key pathological features of depression, including neurotransmitter imbalance and impaired synaptic plasticity.

For Cellular Component (CC), enriched terms indicated localisation to the synaptic membrane, postsynaptic structures, and membrane rafts—suggesting that the corresponding genes may contribute to neuronal receptor assembly and signal-transduction complexes.In molecular functions (MF), enrichment was observed in neurotransmitter receptor activity, G-protein-coupled receptor binding, and ion channel activity, emphasising classical neuronal regulatory mechanisms. KEGG pathway analysis (Figure 3E) supported these results, identifying significant enrichment in neuroactive ligand–receptor interactions, serotonergic synapse, cholinergic synapse, and the cAMP signalling pathway—all of which play key roles in mood regulation, stress response, and neuroinflammation. Notably, “neuroactive ligand–receptor interaction” exhibited the highest enrichment score, suggesting that modulation of multiple neurotransmitter systems may underpin the antidepressant potential of essential oils.The PPI network further identified major hub proteins, including IL6, STAT3, AKT1, MAPK1, MAPK3, MTOR, ESR1, and APP—nodes exhibiting high centrality. Collectively, these findings illuminate interconnected mechanisms involving neurotransmitter signalling, neuroplasticity, and inflammatory regulation.

Although prior studies have predominantly emphasised neurotransmitter regulation and anti-inflammatory effects, the present analysis highlights several promising yet underexplored mechanistic avenues.

First, membrane microdomains (e.g., lipid rafts), which were significantly enriched in the GO-CC analysis, serve as critical platforms for GPCRs, ion channels, and neurotransmitter receptors. Essential-oil metabolites—typically lipophilic and volatile small molecules—may modulate membrane fluidity and lipid-raft stability; however, this potential mechanism has received limited attention in antidepressant research. Second, the HIF-1 signalling pathway, significantly enriched in our KEGG analysis, has rarely been discussed in essential-oil studies. This pathway is implicated in neuroinflammation, mitochondrial function, and neurogenesis, and is closely related to stress adaptation and metabolic regulation (Sharma et al., 2023; Lee et al., 2021). It has also gained traction in depression research over the past 5 years (Gong et al., 2025; Li et al., 2020; Shi et al., 2023), suggesting that essential oils may exert part of their effects via modulation of oxidative stress and cerebral metabolism. Third, the identification of ESR1 as a PPI hub provides novel insights into the molecular connections between “essential oils–hormonal regulation–mood improvement.” While mood modulation is a frequently reported benefit of essential oils, their potential influence on oestrogenic activity or hormone receptor signalling in the brain remains insufficiently explored.

#### 3.4.3 Supportive evidence from computational molecular docking studies for the antidepressant mechanisms of essential oils

In addition to behavioral and biochemical evidence, computational molecular docking studies have increasingly become an important complementary approach for elucidating the antidepressant mechanisms of plant-derived essential oils. By simulating ligand–receptor interactions at the atomic and structural levels, molecular docking provides quantifiable theoretical support for potential binding relationships between chemically characterized metabolites of essential oils and depression-related molecular targets. We conducted a literature survey on several common major metabolites mentioned in the preceding sections and found that, within classical monoaminergic neurotransmitter regulatory pathways associated with depression, a number of essential oil metabolites exhibit noteworthy structural features in molecular docking studies.

Among these, methyl eugenol demonstrated a relatively high binding propensity toward the dopamine D1 receptor and the α1-adrenergic receptor. Its aromatic ring structure is capable of forming stable interactions with hydrophobic and aromatic residues within the receptor active sites (Oliveira et al., 2023). This binding pattern theoretically supports its potential involvement in modulating catecholaminergic signaling pathways associated with anhedonia and psychomotor retardation. Complementary to this finding, linalyl acetate was predicted to interact simultaneously with the serotonin transporter (SERT), the 5-HT1A receptor, and the dopamine D2 receptor (Avram et al., 2021). In addition, limonene, a representative metabolite of Citrus essential oils, exhibited stable binding conformations with SERT in certain docking models. Its interaction characteristics support the hypothesis that limonene may influence synaptic serotonin levels by modulating the 5-HT reuptake process, which is partially consistent with its behavioral effects observed in stress-related animal models (Fonseca et al., 2023).

Beyond the monoaminergic system, certain terpene metabolites have also shown potential value in regulating the balance between excitatory and inhibitory neurotransmission. At the level of inhibitory pathways, linalool exists as two enantiomers, (R)-(−)-linalool and (S)-(+)-linalool, which may exert sedative, anxiolytic, and antidepressant-like effects through actions on targets such as the GABA_*A*_ receptor, the 5-HT_*1A*_receptor, and the 5-HT_3_ receptor. Studies have demonstrated that its anxiolytic effects can be blocked by the GABA_*A*_ receptor antagonist flumazenil, and that linalool exhibits moderate inhibitory activity against the 5-HT_3_ receptor *in vitro*. Moreover, linalool has shown competitive inhibition of glutamate binding in rat cortical membranes *in vitro*, suggesting a potential modulatory effect on the glutamatergic system, including NMDA receptor function, thereby influencing synaptic plasticity (Weston-Green et al., 2021). Given the critical role of NMDA receptors in the rapid antidepressant effects identified in recent years, this finding provides a promising lead for further validation of its neuromodulatory potential.

A comprehensive review and molecular docking study focusing on plant essential oils with antidepressant activity from 2012 to 2022 systematically provided binding energy data for multiple major volatile metabolites and key molecular targets. This work not only confirmed the binding of d-limonene and linalool enantiomers to the serotonin transporter, but also revealed potential binding modes and affinity differences of various monoterpenes, such as α-pinene and γ-terpinene, as well as sesquiterpenes including (−)-β-caryophyllene and patchoulol, toward monoamine transporters or receptors. These findings offer more comprehensive computational simulation evidence for understanding the synergistic, multi-metabolite, and multi-target actions of essential oils (Fonseca et al., 2023). Nevertheless, it must be emphasized that molecular docking is inherently a theoretical prediction tool based on physicochemical principles, and its results cannot be directly equated with *in vivo* pharmacological efficacy or target occupancy. Any translational application, including intranasal delivery, must be founded on rigorous *in vivo* safety and pharmacokinetic validation. Accordingly, these computational data should be regarded as mechanistic clues linking chemical composition to biological effects, providing directional guidance for subsequent experimental studies and target validation efforts.

## 4 Advances in intranasal delivery of botanical essential oils

### 4.1 Bottlenecks in nose-to-brain delivery and technical countermeasures

As an emerging drug administration route, intranasal delivery has demonstrated substantial potential for treating neurological disorders such as depression. By bypassing the blood–brain barrier and transporting drugs directly to the central nervous system (CNS) via the olfactory and trigeminal nerve pathways, this approach avoids hepatic first-pass metabolism, improves bioavailability, and offers advantages including non-invasiveness, ease of administration, and high patient compliance. Despite these benefits, the nasal cavity presents several physiological and anatomical barriers. First, rapid mucociliary clearance drastically limits drug residence time at absorption sites. Second, nasal secretions contain a variety of metabolic enzymes that create a highly degradative biochemical environment; these enzymes can degrade macromolecular therapeutics such as proteins and peptides. Third, the anatomical constraints of the nasal cavity and the epithelial barrier impose physical limitations: the restricted nasal cavity volume limits the dosage that can be delivered, and the olfactory region available for CNS entry is relatively small. Moreover, epithelial cells and their tight junctions hinder the paracellular permeation of macromolecules, substantially reducing the efficiency of CNS drug delivery (Bonferoni et al., 2019; Forbes et al., 2020; Kel et al., 2022). To overcome these bottlenecks, researchers have developed several advanced drug delivery strategies, including nanocarrier systems, mucoadhesion-enhancing technologies, and ligand-based targeting modifications (Babu et al., 2023; Singh and Shukla, 2023; Zha et al., 2022; Li et al., 2022).

Among the various nanocarriers, lipid-based nanoparticles—such as solid lipid nanoparticles (SLNs) and nanostructured lipid carriers (NLCs)—have attracted considerable attention due to their excellent biocompatibility, high drug-loading capacity, and favourable stability, with NLCs providing improved resistance to drug leakage (Zheng et al., 2024). Nanoemulsions, as colloidal dispersion systems with high surface-area-to-volume ratios and ultrafine droplet sizes (typically 100–300 nm), efficiently encapsulate hydrophobic drugs and markedly improve their dissolution rates and stability, making them highly promising platforms for nose-to-brain delivery (Bonferoni et al., 2019). Chitosan, a natural cationic polysaccharide, is widely regarded as an ideal material for nasal-to-brain delivery owing to its excellent biocompatibility, mucoadhesive properties, and permeability-enhancing effects. Its protonated amino groups interact with the mucosal layer to prolong drug retention and can reversibly open epithelial tight junctions, thereby facilitating trans-mucosal transport. Evidence shows that chitosan-modified nanoparticles substantially increase brain drug concentrations and achieve superior brain-targeting efficiency in Parkinson’s and Alzheimer’s disease models compared with conventional formulations (Popescu et al., 2020; Bhattamisra et al., 2020; Yu et al., 2019).

Regarding functional modifications, targeted ligands and stealth strategies further enhance delivery efficiency. Surface modification with specific ligands enables nanocarriers to selectively recognise receptors expressed on olfactory or trigeminal epithelial cells, thereby improving transcellular transport. Meanwhile, stealth strategies—such as polyethylene glycol (PEG)ylation—reduce immunogenic recognition and clearance, prolong systemic circulation time, and increase brain penetration efficiency (Cui et al., 2019; Tian et al., 2025).

### 4.2 Applications of delivery systems for botanical essential oils

In recent years, advanced formulation and delivery technologies have been increasingly applied to botanical essential oils to enhance their stability, bioavailability, and brain-targeting efficiency. For instance, eugenol—the principal bioactive metabolite of clove oil—and ferulic acid methyl ester (Fer-Me) have been co-encapsulated in chitosan oleate–based nanoemulsions, which possess excellent stability and mucoadhesive properties. Animal studies demonstrate that, following intranasal administration, Fer-Me achieves approximately 30-fold higher brain distribution efficiency compared with intravenous administration, while significantly prolonging the cerebral retention of eugenol (Botti et al., 2025).

Research on Lavandula essential oils further highlights the importance of carrier type in modulating CNS pharmacological activity. Nanoemulsions prepared from Lavandula dentata and Lavandula angustifolia essential oils were incorporated into carbomer-based nanogels exhibiting favourable viscosity and pH. These properties extend drug residence time in the nasal cavity, thereby improving absorption and enhancing CNS bioavailability (Nedel et al., 2025). Plant-derived nanovesicles have also emerged as a promising platform for intranasal delivery of neuroactive essential oils. Studies show that grapefruit-derived nanovesicles administered intranasally can cross the blood–brain barrier and deliver encapsulated microRNAs or essential oil metabolites directly to brain tissue. Animal experiments demonstrate effective inhibition of glioma growth and prolonged survival; nanovesicles loaded with lavender or rosemary essential oils exhibit anxiolytic, antidepressant, and cognition-enhancing effects (Zuzarte et al., 2022). In addition, solid lipid nanocarriers (NLCs) formulated using liquid lipids such as olive oil, castor oil, and lavender oil have been explored for intranasal essential oil delivery. Studies report that olive oil–based NLCs, when combined with donepezil in an Alzheimer’s disease mouse model, significantly reduce cerebral β-amyloid burden; castor oil–based systems display notable analgesic and anti-inflammatory activities; and lavender oil–based NLCs exhibit anxiolytic and anti-inflammatory effects, contributing to improved neuroinflammatory status (Tekade et al., 2021).

## 5 Critical appraisal of evidence and future directions

### 5.1 Reliability and clinical relevance of the current research findings

At present, first-line clinical antidepressant therapies are predominantly administered as oral formulations, such as selective serotonin reuptake inhibitors (SSRIs) and serotonin–norepinephrine reuptake inhibitors (SNRIs). Their therapeutic efficacy is generally constrained by first-pass metabolism and the blood–brain barrier (BBB), resulting in delayed onset of action and a relatively high incidence of systemic adverse effects. Against this backdrop, the intranasal route of administration has been well validated in the field of synthetic drugs. For example, intranasal esketamine has demonstrated onset of antidepressant effects within hours in treatment-resistant depression (Bahr et al., 2019), indicating that non-oral delivery strategies may offer distinct advantages in enhancing central nervous system (CNS) drug delivery efficiency.

The intranasal delivery of plant-derived essential oils shows a certain degree of physicochemical compatibility with this route. A systematic review encompassing 40 studies reported that intranasally administered essential oils consistently exhibited antidepressant-like effects across multiple rodent models of depression. Robust behavioral outcomes obtained in established depression models constitute the most reliable evidence, while molecular-level investigations further support the notion that essential oils exert their effects through multi-metabolite, multi-target mechanisms. Specifically, they appear to modulate multiple neurotransmitter pathways, including 5-HT, dopamine (DA), and norepinephrine (NE), rather than acting on a single receptor target.

Importantly, the clinical feasibility of plant essential oils has already been partially validated. The most representative example is Silexan, a standardized lavender oil preparation approved in Germany. Multiple randomized, double-blind, controlled trials have demonstrated that Silexan exerts consistent efficacy in alleviating anxiety and related affective symptoms, and within certain ranges shows a trend toward non-inferiority compared with benzodiazepines or selective serotonin reuptake inhibitors. Moreover, preliminary results from a recent multicenter Phase III clinical trial suggest that Silexan is superior to placebo in patients with major depressive disorder (MDD) and exhibits non-inferiority when compared with sertraline (Kasper and Eckert, 2025).

Beyond oral formulations, advanced delivery systems for other volatile natural products have also entered the stage of clinical validation. For instance, a bergamot essential oil formulation based on solid lipid nanoparticle carriers (NanoBEO) has been evaluated in small-scale clinical studies for the management of agitation and pain in patients with dementia. The results indicated beneficial effects without the observation of adverse reactions typically associated with conventional psychotropic medications. Such studies suggest that formulation optimization can, to some extent, overcome challenges associated with essential oil metabolites, including photosensitivity and odor-related blinding issues, thereby improving the feasibility and reliability of clinical trial design (Scuteri et al., 2024).

In addition to the intranasal route, transdermal patches represent another non-oral delivery strategy that has achieved clinical translation. A representative example is the selegiline transdermal system, which enables sustained drug release, effectively bypasses first-pass metabolism, and improves drug tolerability (Kim et al., 2025). Previous studies have also successfully formulated lavender essential oil into transdermal patches (Fathima et al., 2024); however, most current investigations primarily employ essential oils as transdermal permeation enhancers rather than as active therapeutic agents. Based on the available evidence, among the various delivery modalities, intranasal administration appears to be the most compatible with the pharmacological characteristics of essential oils. Nevertheless, its true clinical value can only be established through direct comparisons with oral and transdermal formulations under standardized conditions, accompanied by systematic evaluation of pharmacokinetics and safety profiles.

In summary, existing evidence indicates that intranasal delivery may theoretically compensate for the delayed onset and first-pass metabolic limitations of oral antidepressants, while offering potential advantages in CNS targeting efficiency. In contrast, alternative delivery strategies such as transdermal patches, although feasible in terms of sustained release and improved tolerability, remain constrained in essential oil applications by factors including transdermal flux, dose control, and the range of applicable indications. At present, there is a lack of systematic comparative studies assessing the relative merits of different delivery technologies in terms of efficacy, safety, and translational potential, representing one of the key unmet needs in this field.

### 5.2 Limitations and challenges

It is noteworthy that, to date, no intranasally delivered essential oil formulation has received broad regulatory approval or been supported by large-scale randomized controlled trials comparable to those for the oral preparation Silexan. This situation is likely attributable to multiple regulatory and technical challenges, including chemistry, manufacturing, and control (CMC) issues arising from batch-to-batch compositional variability; interference of the strong intrinsic odor of essential oils with double-blind study designs; and stringent requirements for inhalation toxicology as well as long-term safety of the nasal mucosa. Compared with small-molecule drugs with well-defined composition and precise dosing, the intrinsic chemical complexity and high volatility of essential oils pose greater difficulties in achieving dose consistency, batch comparability, and long-term local safety evaluation, thereby limiting the overall strength of evidence and regulatory acceptance.

From a biopharmaceutics perspective, the inherent physiological defense mechanisms of the nasal cavity constitute another major challenge. The nasal mucosa is equipped with an active mucociliary clearance system, which, while serving as a protective barrier for the respiratory tract, results in extremely short residence times of administered substances on the olfactory epithelium, thereby markedly restricting drug absorption and the efficiency of nose-to-brain transport via neural pathways. In parallel, substantial interindividual variability in nasal anatomy—such as turbinate size and ventilation status—can lead to pronounced fluctuations in drug deposition sites and absorption efficiency. This factor is often overlooked in standardized animal models but may result in poor reproducibility of therapeutic outcomes in clinical settings (Xu et al., 2024).

From the standpoint of preclinical research, although intranasally administered essential oils have demonstrated certain potential in models of mood disorders, the existing body of evidence remains constrained by multiple methodological limitations and translational barriers. These are mainly concentrated in suboptimal study design and control systems, lack of standardization in dosing procedures, high heterogeneity in composition and dosage, and insufficient systematic safety data. Collectively, these issues undermine the comparability of findings across studies and hinder accurate assessment of their true therapeutic efficacy.

First, with respect to study design and benchmark controls, the current literature generally lacks rigorous and clearly defined comparative frameworks. Some animal studies include only a disease model group, an essential oil intervention group, and a blank control group, without a positive control using a standard antidepressant, making it difficult to assess model sensitivity and the true magnitude of the essential oil effect. Even in studies that incorporate control groups, the specific treatments applied to controls are often insufficiently described. For instance, when animals in the essential oil inhalation group receive intervention, it is frequently unclear whether control animals are exposed to a vehicle odor under identical conditions, merely placed in an enclosed environment without olfactory stimulation, or maintained under normal housing conditions without any specific intervention. The absence of such critical methodological details makes it difficult to disentangle model-related effects from treatment-specific effects, potentially confounding interpretation of the purported actions of essential oils. Similar issues are evident in clinical or quasi-clinical studies, where “standard care” or “placebo odor” is often used as the sole comparator, without parallel comparison with first-line antidepressant medications or evidence-based psychotherapeutic interventions, thereby limiting the clinical contextualization of the findings.

Second, insufficient standardization of administration procedures represents another major limitation. Although most studies report the use of nebulization or inhalation devices, key parameters such as airflow velocity, nebulization efficiency, and exposure volume are often incompletely described. Moreover, under group housing or close-proximity exposure conditions, diffusion of volatile metabolites may occur across experimental groups, leading to actual exposure concentrations that deviate from intended levels; whether measures were taken to prevent intergroup cross-contamination is rarely specified. Importantly, although nasal instillation theoretically allows for relatively precise dosing, in practice animals frequently exhibit stress-related behaviors such as sneezing or head shaking, which can result in loss of the administered solution and introduce substantial dosing variability. Fundamentally, the lack of authoritative operational guidelines for intranasal administration of essential oils in rodents objectively constrains cross-study comparability.

In addition, unlike synthetic drugs with defined chemical entities, the chemical composition of essential oils is influenced by multiple factors, including botanical source, extraction methods, and storage conditions. Consequently, the relative abundance of active metabolites and potential toxic metabolites may fluctuate, increasing the difficulty of quality control. This inherent compositional variability, together with dose-dependent risk profiles, constitutes an additional challenge. Animal studies have suggested that essential oils may exert anxiolytic or antidepressant-like effects at low doses, whereas higher doses may lead to excessive accumulation of lipophilic metabolites in brain tissue, resulting in overstimulation of the nervous system or functional imbalance (Aponso et al., 2020), Thus, the therapeutic window appears to be highly dependent on moderate dosing and controlled exposure. However, systematic toxicological evaluations are notably insufficient in the current literature; most studies are limited to preliminary histological observations, while acute, subchronic, and long-term repeated-dose toxicity assessments are rarely conducted in a systematic manner. *In vitro* studies have demonstrated that lavender oil, tea tree oil, and similar essential oils can induce marked, concentration-dependent cytotoxicity in nasal and pulmonary epithelial cells at higher concentrations, and direct inhalation at elevated levels may also cause ocular or skin irritation (Wojtunik-Kulesza, 2022; Yap et al., 2021). Furthermore, we note that some studies include solubilizing agents in addition to saline when preparing essential oil formulations; these excipients themselves may damage the nasal mucosal structure, further increasing uncertainty regarding the safety of intranasal application (Zhao et al., 2004). Therefore, studies involving direct intranasal instillation warrant particular attention to potential effects on nasal mucosal morphology, especially given that nasal inflammation has been recognized as one of the important contributing factors to the pathogenesis of depression (Wang et al., 2021).

From a regulatory and pharmacokinetic perspective, existing regulatory frameworks are largely based on data derived from oral or transdermal exposure and do not adequately account for the unique risk profiles associated with inhalation routes and nose-to-brain transport. Moreover, among the studies currently available, there is a conspicuous lack of systematic pharmacokinetic investigations addressing the distribution, accumulation, and clearance of essential oil metabolites within brain tissue. Taken together, these considerations indicate that the existing literature is more appropriately regarded as exploratory evidence that highlights research potential and key translational barriers, rather than as robust support for claims of clinical equivalence or superiority. This underscores the fundamental need for future studies to place greater emphasis on standardized study design, rigorous dose control, and comprehensive safety evaluation.

### 5.3 Recommendations for future research

Based on the existing evidence, we believe that future research should move beyond the basic question of “whether it is effective” and place greater emphasis on systematic investigation of several core issues, including the specificity of underlying mechanisms of action, the reliability of preclinical evaluations, the controllability of dose–exposure relationships, and the safety margins associated with long-term use. In parallel, efforts should be made to promote international standardization of study design and reporting. Frameworks such as the ConPhyMP guidelines may be adopted, the core requirements of which include accurate identification and traceability of plant materials; detailed reporting of extraction and preparation procedures; chemical characterization using appropriate fingerprinting methods such as GC–MS; and, wherever possible, quantitative analysis of key metabolites (Heinrich et al., 2022) Adherence to such guidelines represents a cornerstone for improving reproducibility and data comparability, and ultimately for accelerating the clinical translation of formulations with well-defined chemical compositions.

At present, it remains difficult to distinguish whether the observed antidepressant-like effects arise from direct central nervous system delivery via the nose-to-brain route, from systemic exposure following absorption into the circulation, or merely from olfactory stimulation *per se*. In light of the available evidence, we consider techniques such as fluorescent labeling, LC–MS/MS, and mass spectrometry imaging (MSI) to be critical analytical tools for quantitatively assessing the spatial distribution and temporal profiles of key active metabolites in relevant regions, including the olfactory bulb, cerebrospinal fluid, and the hippocampus (Oguro et al., 2025; Wu et al., 2025; Wang et al., 2025). Only through rigorous comparisons across different routes of administration can empirical support be generated for the hypothesis that intranasal delivery confers superior central efficiency, rather than relying solely on theoretical inference.

Second, there is a clear need to enhance the level of standardization in preclinical efficacy evaluation systems. Future study designs should routinely incorporate first-line clinical antidepressants, such as fluoxetine or imipramine, as positive controls; investigators may refer to Table 2 for selection according to specific experimental objectives. Although these drugs are typically administered via oral gavage or intraperitoneal injection, rather than intranasally as with essential oils, they are indispensable for validating the sensitivity and responsiveness of depression models. Their inclusion not only helps to exclude false-positive findings, but also enables a more intuitive assessment of the relative efficacy and onset of action of intranasally administered essential oils compared with established first-line treatments.

In addition, when designing repeated-dose toxicity studies for intranasal essential oil administration, a stepwise, tiered approach is recommended. Early screening studies (14 or 28 days) should focus on identifying toxicity signals and exploring dose–response relationships, thereby informing subsequent investigations. In contrast, pivotal studies intended to support clinical translation should adopt a 90-day subchronic duration to systematically evaluate cumulative toxicity and to establish the no-observed-adverse-effect level (NOAEL). With respect to dose selection, the highest-dose group should be defined by the induction of clear but mild toxicity—such as inhibition of body weight gain of ≤10%—while avoiding excessive tissue damage (Sewell et al., 2022), in order to meet fundamental regulatory requirements for preclinical safety evaluation of new therapeutics. Regarding toxicological assessment, we recommend that, in addition to conventional systemic endpoints, particular attention be paid to local tolerability and central nervous system safety. Given the pronounced lipophilicity of many essential oil metabolites and their potential for tissue accumulation, safety evaluation should not be limited to gross parameters such as body weight or organ coefficients, but should explicitly address risk dimensions unique to intranasal delivery. For example, systematic histopathological examination of the olfactory epithelium and nasal cavity structures should be performed; where feasible, scanning electron microscopy may be employed to precisely assess the ultrastructural integrity of olfactory cilia. Furthermore, measurement of inflammatory cytokines in nasal lavage fluid, such as TNF-α and IL-6, together with analysis of molecular markers in olfactory bulb tissue, can help to preliminarily exclude local inflammatory responses and potential central neurotoxicity. This tiered evaluation strategy facilitates early delineation of safety boundaries, minimizes the risks of membrane toxicity and central neurotoxicity, and provides a more robust safety foundation for subsequent mechanistic studies and clinical translation.

Finally, we consider innovation in formulation technologies and optimization of application modalities to be pivotal drivers of translational progress. Advanced delivery systems, including nanoencapsulation, nanoemulsions, and *in situ* gelling formulations, may improve the stability of volatile metabolites, enhance dose controllability, and reduce the risk of nonspecific membrane toxicity. Beyond purely technological advances, researchers may also explore patient-centered, innovative modes of application. While conventional medical-grade nebulization allows for precise dosing, its dependence on specialized equipment and its overtly “medicalized” nature may compromise long-term patient adherence. By contrast, olfactory-based interventions—such as therapeutic perfumes—represent a highly promising approach for managing mild to moderate depressive symptoms or subclinical affective disturbances. Objectively, such application forms possess a distinct scientific rationale, leveraging the direct modulatory influence of olfactory pathways on the limbic system to achieve mood regulation through sustained low-dose environmental exposure or on-demand olfactory stimulation. Should future studies provide rigorous randomized controlled trial (RCT) evidence comparable to that available for Silexan, these “lifestyle-integrated” interventions could offer not only a noninvasive treatment option with manageable adverse effects, but, more importantly, could effectively mitigate the social stigma associated with psychiatric treatment by embedding therapeutic behavior within everyday grooming practices, thereby maximizing patient adherence and quality of life.

## 6 Summary

Overall, intranasal administration of botanical essential oils represents a highly promising and innovative approach for the treatment of depression. Their volatility and lipophilicity confer favorable properties for nasal absorption and brain targeting, enabling modulation of multiple pathological processes—such as neurotransmitter systems, hypothalamic–pituitary–adrenal (HPA) axis function, neuroinflammation, and oxidative stress—through multi-target mechanisms. In theory, this strategy may circumvent key limitations of conventional oral antidepressants, including delayed onset of action and reliance on single-target pharmacology.

Despite these encouraging prospects, the field remains at an exploratory stage, and several critical bottlenecks hinder clinical translation. First, insufficient standardization in study design and reporting compromises the comparability and reproducibility of findings. Second, systematic safety evidence, particularly long-term toxicological data, is markedly lacking. Third, as complex natural products, essential oils inherently present challenges related to batch-to-batch chemical consistency and quality control. Addressing these limitations will require concerted efforts to standardize research paradigms, advance translational delivery technologies, and establish comprehensive, end-to-end quality regulatory frameworks spanning from raw material sourcing to clinical application. Only once these foundational elements are firmly in place can the therapeutic potential of intranasal essential oil interventions be reliably validated and ultimately translated into clinical practice.

In summary, plant-derived volatile oils, by virtue of their multi-target pharmacological actions and the unique advantages of the nose-to-brain pathway, offer a novel conceptual framework for developing faster-acting and better-tolerated strategies for the management of psychiatric disorders. Their promise warrants continued investigation within rigorous and well-controlled research frameworks.

## Data Availability

The original contributions presented in the study are included in the article/supplementary material, further inquiries can be directed to the corresponding authors.
